# Effects of single-modality sensory stimulation on brain function in patients with prolonged disorders of consciousness: a near-infrared spectroscopy-based study

**DOI:** 10.3389/fneur.2026.1884688

**Published:** 2026-09-29

**Authors:** Juan Zhang, Bin Zhou, Huifang Tian, Tianyi Ma, Siyan Cai, Xuyan Ren, Yingjie Fan, Guodong Zhang, Min Su

**Affiliations:** 1 Department of Physical Medicine and Rehabilitation, The Fourth Affliated Hospital of Soochow University, Suzhou, China; 2 Department of Physical Medicine and Rehabilitation, Suzhou Hospital, Xiyuan Hospital of China Academy of Chinese Medical Sciences, Suzhou, China; 3 Department of General Surgery, Suzhou TCM Hospital Affiliated to Nanjing University of Chinese Medicine, Suzhou, China; 4 Institute of Rehabilitation, Soochow University, Suzhou, China; 5 Department of Physical Medicine and Rehabilitation, Suzhou TCM Hospital Affiliated to Nanjing University of Chinese Medicine, Suzhou, China

**Keywords:** brain function, disorders of consciousness, functional near-infrared spectroscopy, neuroimaging, sensory stimulation

## Abstract

**Objective:**

To explore the effects of four single-modality sensory stimulations on the brain functional networks in patients with prolonged disorders of consciousness (pDOC), and to clarify the correlation between different sensory stimulations and the improvement of patients’ consciousness level, so as to provide references for clinical treatment and rehabilitation of pDOC patients.

**Methods:**

A prospective, multicenter, randomized controlled trial design was adopted. A total of 57 patients with pDOC were enrolled and divided into visual stimulation group, auditory stimulation group, olfactory-gustatory stimulation group, somatosensory-nocieptive stimulation group, and control group. The intervention duration of each group was 15 min. Behavioral assessments were performed using the Coma Recovery Scale-Revised (CRS-R) and Glasgow Coma Scale (GCS). Combined with functional near-infrared spectroscopy (fNIRS), hemodynamic parameters and brain functional connectivity were detected. Changes in brain function and consciousness level before and after different sensory stimulation interventions were evaluated.

**Results:**

Visual, auditory and olfactory-gustatory stimulations induced significant and robust alterations in cerebral activity across most detected channels (*p* < 0.001), while the cerebral response to somatosensory-nocieptive stimulation was relatively limited (*p* < 0.05). At the channel activation level, viewing family photographs in visual stimulation, light music, personalized music and family voice calling in auditory stimulation, and acetic acid stimulation in olfactory-gustatory stimulation significantly activated the corresponding brain regions (*p* < 0.05). At the brain functional connectivity level, viewing family photographs markedly enhanced functional connectivity within visual-related brain regions (*p* < 0.05); ethanol stimulation improved functional connectivity of olfactory-gustatory brain regions (*p* < 0.05).

**Conclusion:**

Single-modality sensory stimulation elicits acute cerebral activation in pDOC patients, which is superior to conventional rehabilitation alone. Visual and auditory stimulations exert significantly better intervention effects than olfactory-gustatory and somatosensory-nocieptive stimulations. Among olfactory-gustatory interventions, acetic acid stimulation produces prominent brain region activation, and ethanol enhances functional connectivity of related brain regions. Both can serve as an effective supplementary intervention for pDOC patients with visual and auditory dysfunction, and possess important value for further research and clinical application in the management of pDOC.

## 1 Introduction

Prolonged disorders of consciousness (pDOC) is an umbrella clinical term defined per international consensus for disorders of consciousness persisting for at least 28 days after severe acute acquired brain injury. It encompasses the vegetative state (VS)/unresponsive wakefulness syndrome (UWS) and the minimally conscious state (MCS), severe neurological syndromes resulting from multiple etiologies including severe traumatic brain injury, stroke, and hypoxic encephalopathy (1, 2). The core clinical feature of pDOC patients is persistent loss or impairment of conscious awareness, which not only imposes a heavy physical, psychological and economic burden on patients’ families, but also poses great challenges to the allocation of social medical resources and clinical ethical decision-making (3–5). At present, the clinical diagnosis of pDOC still relies mainly on behavioral assessment tools such as the Coma Recovery Scale-Revised (CRS-R) (6). However, such scales have inherent limitations, including high misdiagnosis rate, obvious dependence on the clinician’s experience, and strong subjectivity (7, 8). Studies have shown that the clinical misdiagnosis rate of pDOC can reach 20–40% when relying solely on behavioral evaluation, which seriously affects the formulation of clinical treatment plans and the judgment of patient prognosis (9–11).

With the rapid development of neuroimaging techniques, functional neuroimaging modalities such as functional magnetic resonance imaging (fMRI), electroencephalography (EEG), and functional near-infrared spectroscopy (fNIRS) have provided objective biomarkers for the diagnosis and prognostic evaluation of pDOC (12–14). Among them, fNIRS possesses the advantages of non-invasiveness, portability, high temporal resolution, and low sensitivity to motion artifacts, and has been widely used in real-time monitoring of cerebral functional activity in pDOC patients (15, 16). By detecting concentration changes of oxyhemoglobin and deoxyhemoglobin in the cerebral cortex, this technique reflects dynamic alterations in cerebral oxygen metabolism, and further enables quantitative evaluation of brain region activation and functional connectivity of neural networks (17–19), offering a novel approach for objective assessment of brain function in pDOC patients.

Currently, there is no specific curative regimen for pDOC in clinical practice. Rehabilitation interventions are dominated by conventional physical therapy, occupational therapy and single-modal sensory stimulation (20, 21). As a promising rehabilitation strategy, multimodal sensory stimulation activates different sensory conduction pathways by delivering standardized visual, auditory, olfactory-gustatory, and somatosensory-nocieptive stimuli, thereby promoting the reconstruction of cerebral functional networks and the recovery of consciousness (22, 23). Cumulative studies have demonstrated that unimodal sensory stimulation (e.g., auditory and visual stimuli) elicits cerebral responses in patients with pDOC, including regional cortical activation within sensory-related brain areas, sensory-evoked electrophysiological waveforms, and detectable haemodynamic fluctuations (24). Nevertheless, the differential effects of distinct sensory modalities on brain function, as well as the neural mechanisms underlying consciousness recovery, remain unclear. In addition, response differences across sensory modalities and the synergistic effect of multisensory integration remain controversial in clinical research (25, 26).

Adopting a prospective, multicenter, randomized controlled trial design, the present study delivered standardized single-modality sensory stimulation interventions to pDOC patients. Real-time changes in cerebral functional activity were monitored using fNIRS, combined with clinical behavioral rating scales. We comprehensively evaluated the effects of different sensory modalities on cerebral functional networks in pDOC patients, clarified the neural correlation between sensory stimulation and consciousness improvement, and explored the optimal stimulation modality and intervention protocol for pDOC patients. This study aims to provide scientific and effective therapeutic strategies for the clinical rehabilitation of pDOC patients.

## 2 Materials and methods

### 2.1 Study design

This study was designed as a prospective, multicenter, randomized controlled trial. Random sequence was generated by a computerized random number generator. Allocation concealment was implemented using sequentially numbered, sealed opaque envelopes. A single-blind design was adopted. Therapists performing interventions were unblinded. Only assessors and statisticians were blinded to group allocation. The protocol was approved by the Medical Ethics Committee of each participating hospital (Approval No. 2024-Ethics-240046). Written informed consent was obtained from the legal authorized representatives of all patients. The study was strictly conducted in accordance with the Consolidated Standards of Reporting Trials (CONSORT) guidelines to ensure scientific rigor, standardization, and reproducibility of the research procedure.

### 2.2 Study participants

Patients with prolonged disorders of consciousness (pDOC) were recruited from three general hospitals between December 2024 and February 2026. Inclusion criteria: ① Diagnosed as vegetative state/unresponsive wakefulness syndrome (VS/UWS) or minimally conscious state (MCS) according to the clinical diagnostic criteria for pDOC, with a disease course of no less than 28 days after brain injury; ② Aged 18–75 years; ③ Stable vital signs without severe complications such as serious infection or intracranial hemorrhage; ④ Able to tolerate sensory stimulation intervention and functional near-infrared spectroscopy (fNIRS) examination. Exclusion criteria: ① Pre-existing severe cognitive impairment, psychiatric disorders, or other neurological diseases before brain injury; ② Severe injury to the face or sensory organs that prevented reception of sensory stimulation; ③ Unstable hemodynamics that precluded completion of the whole intervention and examination; ④ Refusal of family cooperation or high risk of loss to follow-up.

A total of 65 patients were initially screened, among whom 8 were excluded due to failure to meet the inclusion criteria or refusal by family members. Finally, 57 pDOC patients were enrolled, including 37 males and 20 females, with an average age of 59.5 ± 11.8 years (range, 32–79 years). Etiologies of brain injury included severe traumatic brain injury (TBI) (17 cases), and stroke (40 cases). Patients were randomly allocated using a random number table into five groups: control group (13 cases), visual stimulation group (12 cases), auditory stimulation group (10 cases), olfactory-gustatory stimulation group (11 cases), and somatosensory-nocieptive stimulation group (11 cases). There were no significant between-group differences in gender, age, etiology, diagnosis, duration(all *p* > 0.05), indicating good baseline comparability (Table 1).

**Table 1:** Baseline demographic and clinical characteristics of patients with prolonged disorders of consciousness across the groups.

Group	*n*	Gender (male/female)	Age (years), median (IQR)	Diagnosis (UWS/MCS)	Etiology (TBI/Stroke)	Duration(months), median (IQR)
Visual stimulation group	12	7/5	59.0 (54.0–65.5)	7/5	9/3	8.5 (5.5–14.0)
Auditory stimulation group	10	7/3	59.5 (56.2–66.8)	6/4	7/3	11.5 (7.8–17.5)
Olfactory-gustatory stimulation group	11	7/4	61.0 (58.0–64.5)	6/5	7/4	9.0 (7.5–15.5)
Somatosensory-nocieptive stimulation group	11	8/3	58.0 (54.0–66.5)	6/5	8/3	9.0 (6.5–12.5)
Control group	13	8/5	61.0 (55.0–65.0)	7/6	9/4	11.0 (7.5–14.5)

TBI, traumatic brain injury; IQR, Interquartile Range; UWS, nresponsive Wakefulness Syndrome; MCS, Minimally Conscious State.

### 2.3 Intervention protocol

All interventions were performed in a quiet, comfortable ward with ambient temperature maintained at 22–26 °C and soft indoor lighting to avoid external interference. Each stimulation block lasted 2 min with a 30-s inter-block interval. The total intervention duration was standardized to 15 min including rest periods. The control group received no additional sensory stimulation; all enrolled patients underwent routine rehabilitation and nursing care throughout the study. Vital signs were monitored throughout stimulation; any adverse reactions (discomfort, agitation, hypertension) led to immediate termination. All chemical stimuli (acetic acid, ethanol) were approved for clinical use and applied at non-irritating concentrations. The detailed intervention protocols for each group were as follows.

#### 2.3.1 Visual stimulation group

Social–emotional stimulation and basic physical stimulation were presented alternately, each lasting 2 min. ① Social–emotional stimulation: High-definition frontal photographs of patients’ immediate relatives, with unified shooting angle, neutral facial expression, and pure white background; ② Basic physical stimulation: Pantone standard color cards with high-contrast red, blue and yellow; taboo colors related to patients’ medical history were excluded (27).

#### 2.3.2 Auditory stimulation group

Social–emotional stimulation, basic physical stimulation, and personalized favorite music were presented alternately, each lasting 2 min. ① Social–emotional stimulation: Standardized voice recordings of family members calling the patient (content: “I am your [family relation], can you hear me?”); ② Basic physical stimulation: Air on the G String by Bach, with a frequency range of 250–4,000 Hz and volume calibrated uniformly to 60 dB SPL (28); ③ Personalized preferred music.

#### 2.3.3 Olfactory-gustatory stimulation group

5% (v/v) acetic acid solution and 40% (v/v) ethanol solution were used as stimulation materials. Nasal olfactory stimulation and lingual gustatory stimulation were applied alternately for 1 min each, with each stimulus lasting 2 min. All operations were performed by professional medical staff to standardize stimulation intensity and application site.

#### 2.3.4 Somatosensory-nocieptive stimulation group

Somatosensory and nocieptive stimulation were delivered alternately, each lasting 2 min. ① Somatosensory stimulation: Semmes–Weinstein monofilaments; ② Noxious stimulation: A 0.5 kg percussion hammer with a fixed falling distance of 10 cm was used to deliver transient mechanical stimulation. Stimulation sites included five bony landmark points bilaterally at the shoulder, elbow, wrist, knee, and ankle. Nocieptive stimulation intensity was calibrated to grade 2–3 pain in healthy volunteers and applied transiently to avoid tissue damage.

### 2.4 Outcome measures

#### 2.4.1 Clinical behavioral assessment

Consciousness level was evaluated before and immediately after intervention using the Coma Recovery Scale-Revised (CRS-R) and Glasgow Coma Scale (GCS). The CRS-R consists of six subscales (auditory, visual, motor, oromotor/verbal, communication, and arousal) with 23 hierarchical items; the total score ranges from 0 to 23, and a higher score indicates a better level of consciousness. The GCS evaluates eye opening, verbal response, and motor response, with a total score of 3–15; a score ≤8 indicates severe consciousness disturbance. All assessments were performed by two rehabilitation physicians with more than 5 years of clinical experience.

#### 2.4.2 fNIRS measurements

Data were acquired using a functional near-infrared spectroscopy (fNIRS) system (Model H01B, Bairuixin Intelligent Technology Co., Ltd., China). The system consisted of 8 light sources (labeled L1–L8) and 8 detectors (labeled D1–D8), arranged in a 4 + 2 × 6 array to form a total of 20 valid recording channels (labeled 1–20). The light sources emitted near-infrared light at wavelengths of 785 nm and 830 nm. The center-to-center distance between adjacent sources and detectors was 3 cm, with a theoretical detection depth of approximately 0.5 cm below the cerebral cortex. The temporal resolution of the system was 0.1 s, enabling real-time monitoring of changes in oxygenated hemoglobin concentration in the medial prefrontal cortex (mPFC) and bilateral primary motor cortices (lM1, rM1). Data preprocessing and analysis were performed using the supporting software of the fNIRS system (RC01B-400S, Bairuixin Intelligent Technology Co., Ltd., China). Temporal Derivative Distribution Repair (TDDR) was applied to identify and remove motion artifacts. A Butterworth bandpass filter with a frequency range of 0.01–0.1 Hz was then used to reduce high-frequency noise and slow signal drift. Detected hemodynamic indicators included oxyhemoglobin concentration, deoxyhemoglobin concentration, and total hemoglobin concentration; the mean relative changes in cerebral oxygenation were calculated. Meanwhile, channel activation and brain functional connectivity analyses were performed. Functional connectivity strength between different brain regions was evaluated by calculating the correlation coefficient of hemoglobin signal changes among channels (Figure 1).

**Figure 1:**
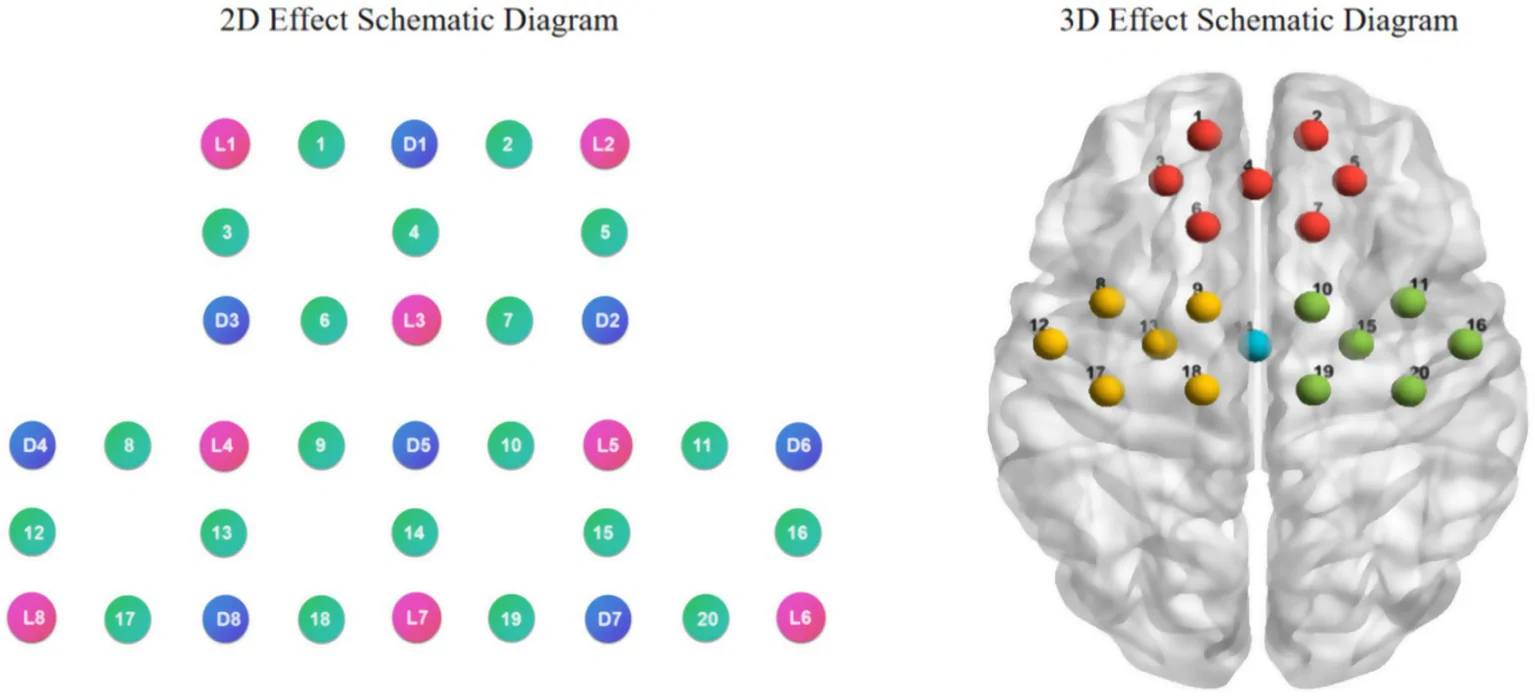
Headcap Acquisition Channel Layout and Brain Region Correspondence Map: Eight light sources (red circles, L1–L8), eight detectors (blue circles, D1–D8), twenty measurement channels (green circles, numbered 1–20). The medial prefrontal cortex (mPFC) region comprises Channels 1, 2, 3, 4, 5, 6, 7; the left primary motor cortex (lM1) region comprises Channels 8, 9, 12, 13, 17, 18; the right primary motor cortex (rM1) region comprises Channels 10, 11, 15, 16, 19, 20.

#### 2.4.3 Evaluation of brain functional activation intensity

Functional near-infrared spectroscopy (fNIRS) was used to acquire time-series signals of oxyhemoglobin (HbO) in each channel. Linear regression analysis was performed on task-state HbO signals to obtain the slope of signal changes (unit: mmol/L·mm), which was adopted to quantify the intensity of cerebral functional response in individual channels. An independent-samples t-test was applied to compare the slope differences between the single-modality sensory stimulation groups and the control group; a *p* < 0.05 was considered statistically significant.

### 2.5 Statistical analysis

Data processing was performed using SPSS 26.0 software, and graph plotting was conducted with GraphPad Prism 9.0. Normally distributed measurement data were expressed as mean ± standard deviation ( $\bar{x}$ ± s). One-way analysis of variance (ANOVA) was used for comparison among multiple groups, and the least significant difference (LSD) method was adopted for pairwise intergroup comparisons. The Kruskal–Wallis H test was employed for non-parametric multiple-group comparisons. Enumeration data were presented as case numbers and constituent ratios, and the chi-square (χ^2^) test was used for intergroup comparison. Pearson correlation analysis was utilized to explore the correlation between cerebral functional indicators and consciousness scale scores. A *p* < 0.05 was defined as statistically significant.

## 3 Results

### 3.1 Comparison of clinical behavioral scores among groups

Baseline CRS-R and GCS scores were well balanced across all groups (*p* > 0.05). Paired t-test showed that post-intervention CRS-R and GCS scores were significantly higher than baseline in all groups (all *p* < 0.001). One-way ANOVA showed significant between-group differences in CRS-R score improvements (*F* = 33.85, *p* < 0.001). *Post hoc* tests indicated that all sensory stimulation groups exhibited significantly greater score improvements than the control group (all *p* < 0.001). Among them, the visual stimulation group achieved the highest improvement, which was significantly superior to the auditory, olfactory-gustatory, and somatosensory-nocieptive stimulation groups (all *p* < 0.05).

A significant intergroup difference was also found in the improvement of GCS scores (*F* = 11.67, *p* < 0.001). The visual stimulation group showed a significantly greater increase than the control group and the somatosensory-nocieptive stimulation group (all *p* < 0.001). The auditory and olfactory-gustatory stimulation groups had significantly larger improvements than the somatosensory-nocieptive stimulation group (all *p* < 0.05). These findings suggest that, as expected, the CRS-R is more sensitive than the GCS for evaluating subtle changes in consciousness induced by interventions (Table 2).

**Table 2:** Comparison of CRS-R and GCS scores before and after intervention in each group ( $\bar{x}$ ±s, points).

Group	*n*	CRS-R (Mean±SD)					GCS (Mean±SD)				
		Before intervention	After intervention	Difference	*t*	*p*	Before intervention	After intervention	Difference	*t*	*P*
Visual stimulation group	12	6.92 ± 0.79	12.00 ± 0.85	5.08 ± 0.51	34.51	<0.001	8.17 ± 0.83	12.25 ± 0.87	4.08 ± 0.51	27.9	<0.001
Auditory stimulation group	10	7.10 ± 0.88	11.70 ± 1.34	4.60 ± 0.70	20.8	<0.001	7.50 ± 0.97	10.80 ± 1.03	3.30 ± 0.82	12.73	<0.001
Olfactory-gustatory stimulation group	11	7.36 ± 1.36	11.73 ± 1.27	4.36 ± 0.81	17.89	<0.001	8.82 ± 0.98	12.00 ± 1.00	3.18 ± 0.40	26.49	<0.001
Somatosensory-nocieptive stimulation group	11	7.64 ± 1.21	11.91 ± 1.04	4.27 ± 0.65	21.91	<0.001	8.73 ± 1.19	11.09 ± 1.38	2.36 ± 0.67	11.7	<0.001
Control group	13	6.54 ± 0.97	9.08 ± 1.04	2.54 ± 0.78	11.43	<0.001	8.69 ± 1.25	11.31 ± 1.25	2.62 ± 0.77	12.24	<0.001

### 3.2 Overall brain functional activation intensity induced by different sensory modalities

Compared with the control group, all sensory stimulation conditions elicited significant activation responses in the prefrontal cortex, with remarkable differences in activation intensity. Visual stimulation (viewing family photographs) produced the strongest and most stable activation, with significant differences observed in all channels (all *p* < 0.05), and markedly lower *p* values in most channels (all *p* < 0.001). Auditory stimulation (preferred music, light music, and family voice) ranked second (all *p* < 0.001). Olfactory-gustatory stimulation (acetic acid and ethanol) yielded a relatively weaker but still significant activation effect (*p* < 0.001). Somatosensory-nociceptive stimulation induced moderate activation, whereas noxious stimulation resulted in the weakest and least stable activation response (*p* < 0.05). Under most experimental conditions, Channels 5, 7, 14 and 15 showed significantly higher t values, indicating that these regions represented the core responsive areas within the prefrontal cortex.

### 3.3 Channel activation characteristics of different sensory modalities

#### 3.3.1 Visual stimulation

Viewing family photographs significantly activated all 20 detection channels (*p* < 0.001 or *p* < 0.01) with generally high *t* values (e.g., Channel 13, *t* = 4.278), suggesting that socioemotional visual stimulation can effectively activate visual-related brain regions. By contrast, viewing color cards exerted no significant activating effect on most channels (*p* > 0.05), with only a few channels approaching the critical level, indicating that basic physical visual stimulation has a limited role in cerebral activation.

#### 3.3.2 Auditory stimulation

Light music, personalized preferred music, and family calling voice all significantly activated all detection channels (*p* < 0.001 or *p* < 0.01) and presented high t values (e.g., light music in Channel 10, *t* = 4.394; personalized music in Channel 9, *t* = 4.005; family voice in Channel 10, *t* = 4.05). The activation intensity induced by personalized music and family voice was slightly higher than that of light music, implying that emotional and individualized auditory stimulation achieves superior cerebral activation.

#### 3.3.3 Olfactory-gustatory stimulation

The 40% (v/v) ethanol solution produced no significant activation in most channels (*p* > 0.05), with only a few channels approaching the threshold at *p* = 0.05. In contrast, the 5% (v/v) acetic acid solution significantly activated the majority of channels (e.g., Channels 1–9 and 13, *p* < 0.001 or *p* < 0.01) with relatively high t values (e.g., Channel 9, t = 3.972), demonstrating that strong odor stimulation can effectively activate olfactory-gustatory associated brain regions.

#### 3.3.4 Somatosensory-nocieptive **stimulation and control group**

Somatosensory-nocieptive stimulation failed to induce significant activation across all detection channels (*p* > 0.05) with generally low *t* values (e.g., Channel 10, *t* = 0.322). Similarly, the control group showed no significant activation in any channel (*p* > 0.05) and maintained low t levels (e.g., Channel 10, *t* = 0.651). The mean relative changes in blood oxygenation across sensory modalities indicated that visual and auditory stimulation elicited the most prominent hemodynamic responses, followed by olfactory-gustatory stimulation (acetic acid). Both somatosensory-nocieptive stimulation and the control group exhibited relatively weak blood oxygenation responses (Figure 2).

**Figure 2:**
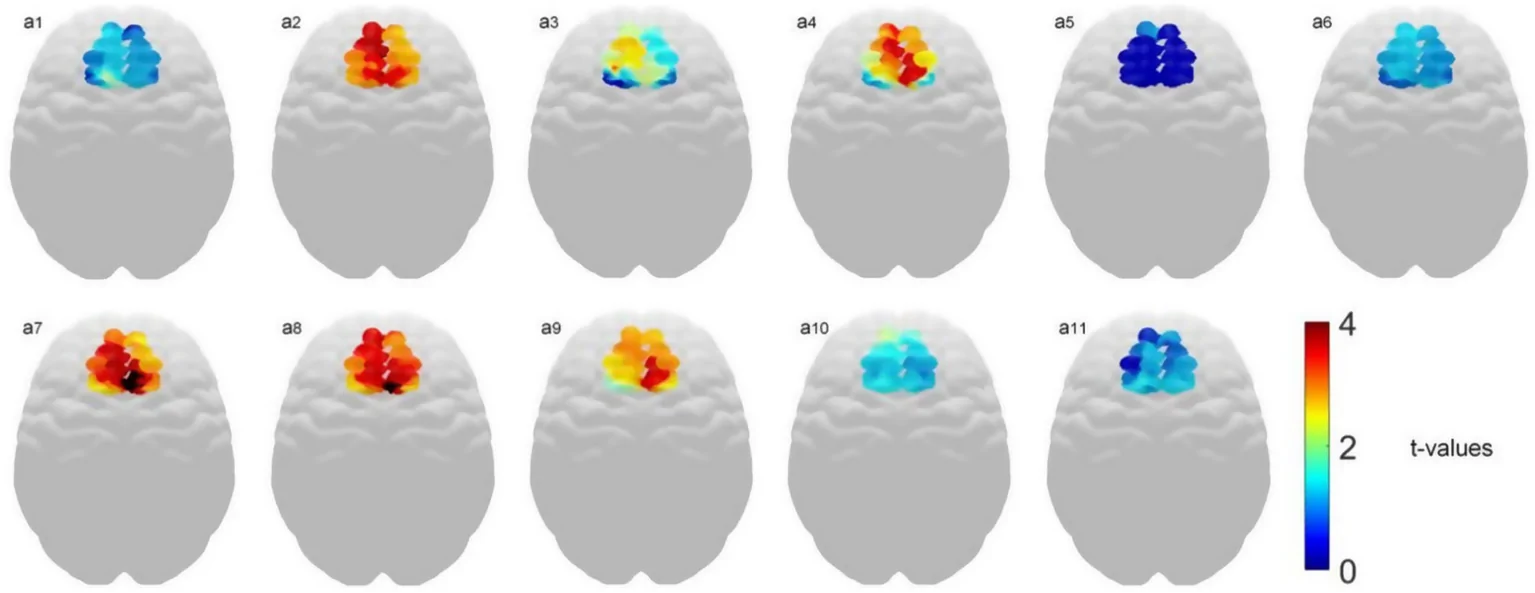
A total of 11 subplots are presented, corresponding to independent samples t-test results comparing each of the 11 stimulation conditions with the control group. (a1-a11, color card viewing vs. Control, family photograph viewing vs. Control, alcohol odor vs. Control, vinegar odor vs. Control, alcohol taste vs. Control, vinegar taste vs. Control, light music vs. Control, patient-preferred music vs. Control, family-recorded voice/music vs. Control, somatosensory vs. Control, nociceptive vs. Control.). The panel shows the 3D brain surface rendering. The color bar (right) represents *t*-values (range: 0–4), where warm colors indicate high *t*-values and strong statistical significance of inter-group differences, while cool colors indicate low t-values and non-significant differences. All significance levels were corrected using the false discovery rate (FDR) procedure, with *p* < 0.05 considered statistically significant.

### 3.4 Characteristics of brain functional connectivity under different sensory modalities

#### 3.4.1 Visual stimulation

Viewing family photographs significantly strengthened functional connectivity within visual-related brain regions. Multiple channel combinations showed high correlation coefficients of hemodynamic signal changes (*p* < 0.05) with elevated t values (e.g., Channels 5–9, *t* = 4.027, *p* < 0.01). By comparison, viewing color cards exerted no significant enhancement on visual functional connectivity; most channel combinations presented marginal *p* values near 0.05 and low t values, indicating a limited facilitating effect on brain network integration.

#### 3.4.2 Auditory stimulation

Light music, personalized music, and family voice calls generally showed weak promotion of functional connectivity in auditory-related brain regions. Only a small number of channel combinations approached the significant level (P ≈ 0.05), without widespread enhancement of functional connectivity. Some channel combinations even exhibited a tendency of decreased connectivity (e.g., Channels 10–11 under light music, *t* = −2.89, *p* = 0.049), suggesting that auditory stimulation yields unstable effects on the integration of auditory brain networks.

#### 3.4.3 Olfactory-gustatory stimulation

The 40% (v/v) ethanol solution significantly enhanced functional connectivity in olfactory-gustatory related brain regions, with statistically significant differences across most channel combinations (*p* < 0.05) and high t values (e.g., Channels 1–5, *t* = 2.212, *p* < 0.01). In contrast, the 5% (v/v) acetic acid solution only reached marginal significance in a single channel combination (Channels 15–19, *t* = 2.052, *p* = 0.05), showing a weak effect on improving functional connectivity.

#### 3.4.4 Somatosensory-nocieptive stimulation and control group

Somatosensory-nocieptive stimulation produced no significant enhancement of brain functional connectivity. Most channel combinations showed *p* values around 0.05 and relatively low t values (e.g., Channels 2–18, *t* = 2.296). Although a few channel combinations in the control group reached statistical significance (*p* < 0.05), the overall significant range was limited. These results indicate that conventional rehabilitation alone has a restricted effect on improving brain network integration in pDOC patients (Figure 3).

**Figure 3:**
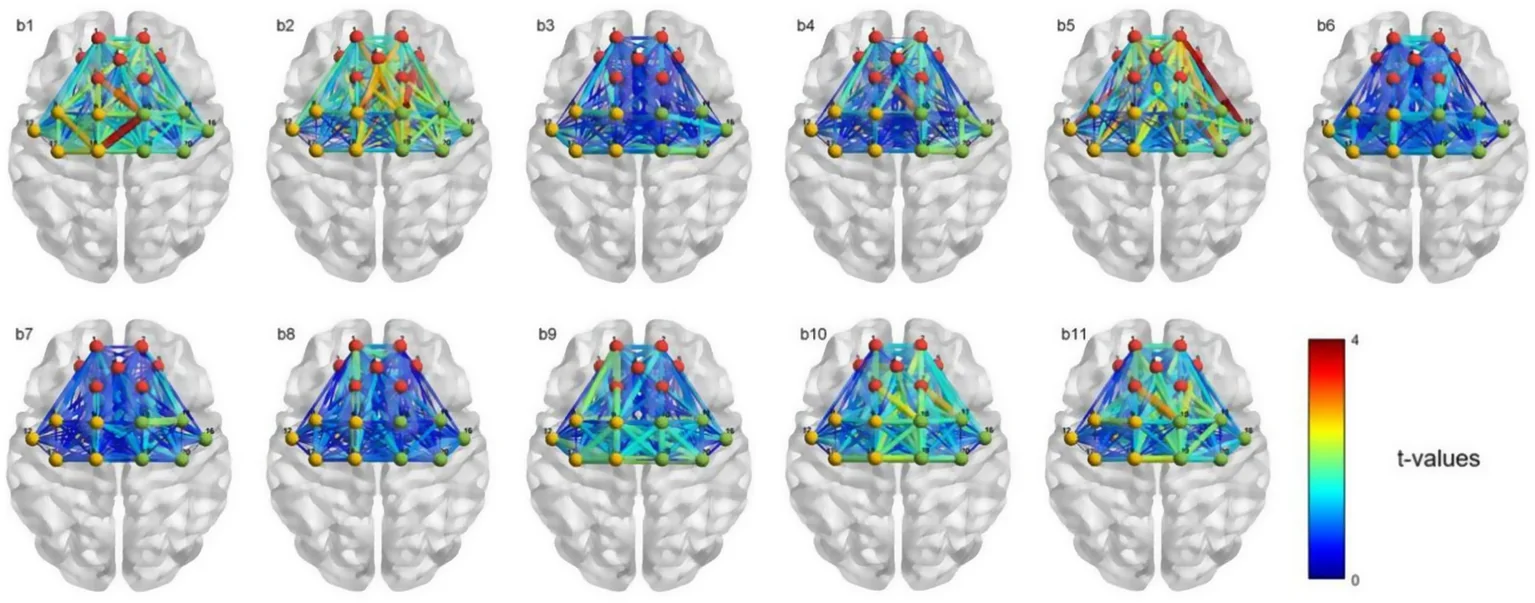
A total of 11 subplots are presented, corresponding to independent samples t-test results comparing each of the 11 stimulation conditions with the control group. (b1-b11, color card viewing vs. Control, family photograph viewing vs. Control, alcohol odor vs. Control, vinegar odor vs. Control, alcohol taste vs. Control, vinegar taste vs. Control, light music vs. Control, patient-preferred music vs. Control, family-recorded voice/music vs. Control, somatosensory vs. Control, nociceptive vs. Control). The panel shows the 3D brain surface rendering. The color bar (right) represents *t*-values (range: 0–4), where warm colors indicate high *t*-values and strong statistical significance of inter-group differences, while cool colors indicate low *t*-values and non-significant differences. All significance levels were corrected using the false discovery rate (FDR) procedure, with *p* < 0.05 considered statistically significant.

## 4 Discussion

### 4.1 Advantages of single-modality sensory stimulation in the treatment of pDOC

The present study observed short-term improvements in consciousness-related scale scores following single-modality sensory stimulation relative to conventional rehabilitation physiotherapy among patients with pDOC, which is consistent with findings from previous studies (20, 29, 30). After intervention, the CRS-R and GCS scores in the four stimulation groups were significantly higher than those in the control group, indicating that sensory stimulation can effectively improve the level of consciousness in pDOC patients. fNIRS results demonstrated that single-modal sensory stimulation induced more pronounced cerebral activity and stronger hemodynamic responses than non-intervention conditions, which is closely associated with the activation of specific sensory conduction pathways in the brain (31–33).

Severe brain injury often damages cerebral functional networks and reduces neuronal activity levels. By providing external neural input, sensory stimulation promotes the release of neurotransmitters such as glutamate and dopamine, enhances neuronal activity in injured brain regions, and further facilitates the reconstruction of cerebral functional networks and the recovery of consciousness (34, 35). The control group only received routine nursing without effective external neural input, resulting in no obvious improvement in cerebral responses or consciousness level. This further emphasizes the necessity of active sensory stimulation intervention for pDOC patients.

### 4.2 Efficacy differences among different sensory modalities for pDOC treatment

Significant differences in therapeutic effects were observed among various sensory stimulation modalities. Classical visual and auditory stimulation achieved markedly better outcomes than olfactory-gustatory and somatosensory-nocieptive stimulation, which may be attributed to the structural and functional characteristics of cerebral sensory processing pathways (36–38). The visual and auditory conduction pathways are relatively well-developed, and related cortical regions including the visual cortex, auditory cortex, and prefrontal cortex retain strong neural plasticity. Even after severe brain injury, these brain regions maintain a certain responsiveness to external stimuli (39–41).

Meanwhile, visual and auditory stimulation are easy to standardize and individualize. Combined with socioemotional factors such as family photographs and family voice recordings, such stimulation can further enhance cerebral activation (42, 43), which constitutes an important reason for their superior intervention efficacy.

The therapeutic effect of olfactory-gustatory stimulation was relatively limited, possibly owing to the relatively simple neural pathways and fewer involved brain regions for olfactory and gustatory processing (44). In addition, olfactory-gustatory function is often impaired in pDOC patients, leading to decreased sensory sensitivity and weakened cerebral responses (31, 45). Nevertheless, this study found that acetic acid stimulation produced prominent cerebral activation, while ethanol stimulation enhanced functional connectivity in related brain regions. Strong odor stimulation can activate the limbic system and the brainstem reticular activating system, thereby elevating the level of cerebral arousal (46). For olfactory-gustatory stimulation: Acetic acid evoked strong cortical activation but minimal enhancement of functional connectivity. This pattern indicates that acetic acid mainly induces transient brain arousal via the brainstem reticular activating system, rather than facilitating long-range neural network integration (47). Previous fNIRS studies have confirmed that acidic stimulation can trigger robust cortical activation in sensory and prefrontal regions. In contrast, 40% ethanol induced weak activation but significantly enhanced regional functional connectivity. This dissociation suggests that ethanol acts as a neuromodulator that modulates synaptic transmission and neural synchrony through GABAergic and dopaminergic pathways, thereby strengthening functional connections within targeted networks without evoking intense hemodynamic responses (48). This finding provides a novel intervention approach for pDOC patients with visual and auditory dysfunction, supporting olfactory-gustatory stimulation especially strong odor stimulation as a complementary rehabilitation strategy.

Somatosensory-nocieptive stimulation showed the poorest therapeutic effect in this study, with no significant cerebral activation or enhanced functional connectivity detected. This may be related to impaired somatosensory conduction pathways, decreased signal transmission ability, and reduced cerebral sensitivity to somatosensory-nocieptive stimulation in pDOC patients (49, 50). Furthermore, the relatively single form and insufficient intensity of the somatosensory-nocieptive protocol adopted in this study (Semmes–Weinstein monofilaments and standardized percussion hammer) may also account for the unsatisfactory outcomes. Future research should optimize stimulation protocols by increasing stimulation frequency, intensity, and diversity to explore more effective somatosensory intervention patterns.

### 4.3 Reinforcing effect of emotional and individualized stimulation on cerebral functional activation

This study revealed that emotional and individualized sensory stimulation induces significantly stronger cerebral activation than basic physical stimulation. In the visual domain, viewing family photographs yielded more prominent cerebral activation and functional connectivity enhancement than viewing color cards. In the auditory domain, family voice calls and personalized music produced higher activation intensity than light music.

Emotional and individualized stimuli can activate emotion-processing brain regions such as the amygdala and hippocampus, forming robust neural correlations with sensory processing areas and thereby enhancing cerebral responsiveness and the integration capacity of brain functional networks (51, 52). Previous studies have also verified that emotional stimulation improves consciousness level more effectively than neutral stimulation in pDOC patients (53, 54). Therefore, individualized sensory stimulation protocols based on family relationships and personal preferences are recommended in clinical rehabilitation to optimize intervention efficacy.

### 4.4 Application value of fNIRS in evaluating the efficacy of single-modality sensory stimulation

As a non-invasive neuroimaging technique, fNIRS plays a crucial role in objective outcome evaluation in this study (45). It enables real-time monitoring of cerebral oxygen metabolism during sensory intervention and quantitatively assesses cerebral activation and functional connectivity through indicators such as mean hemodynamic changes, t values, and correlation coefficients.

Compared with other neuroimaging modalities such as fMRI, fNIRS features portability and low susceptibility to motion artifacts, making it more suitable for long-term monitoring in pDOC patients with limited motor function (16, 55). Moreover, fNIRS does not require active patient cooperation and can be performed under natural resting conditions, which fits the clinical characteristics of impaired consciousness in pDOC patients (56). This study confirms that fNIRS serves as an objective and effective tool for evaluating the therapeutic efficacy of single-modality sensory stimulation, providing scientific evidence for the formulation and optimization of clinical intervention protocols.

### 4.5 Limitations and future perspectives

Several limitations of this study should be acknowledged: ① The relatively small sample size may introduce selection bias; further large-sample studies are required to validate the present findings. ② This study only explored the effects of single-modal sensory stimulation; the synergistic effects of multisensory integrated stimulation on brain function and consciousness recovery remain unclear. ③ The long-term therapeutic effects and prognostic impact of single-modality sensory stimulation require further long-term follow-up observation. ④ fNIRS is limited to measuring hemodynamic signals within the superficial prefrontal and motor cortices. We will combine functional magnetic resonance imaging in future work for cross-modal integration to further extend its detection coverage.

On the basis of the current results, future research should expand the sample size and conduct long-term follow-up to explore the long-term efficacy and neural mechanisms of multisensory intervention. Various integrated single-modality sensory stimulation protocols should be designed to clarify the synergistic effects among different sensory modalities. Intervention strategies can be further optimized according to pDOC subtypes and individual patient characteristics, so as to provide more personalized and effective rehabilitation regimens.

In addition, combining fNIRS with other neuroimaging (fMRI) and electrophysiological techniques to establish a comprehensive brain function evaluation system will help improve the accuracy of clinical diagnosis and therapeutic effect assessment for pDOC.

## 5 Conclusion

Single-modality sensory stimulation can effectively improve cerebral functional activity and consciousness level in pDOC patients, with superior efficacy compared with conventional rehabilitation physiotherapy. Brain functional responses to sensory stimulation exhibit obvious heterogeneity across modalities. Visual and auditory stimulation significantly activate corresponding brain regions and facilitate consciousness recovery, serving as core intervention approaches for pDOC. Olfactory-gustatory stimulation induces notable cerebral activation and enhances regional functional connectivity, and can be adopted as an effective supplementary intervention for pDOC patients with visual and auditory dysfunction. Emotional and individualized sensory stimulation can amplify cerebral activation effects and is more applicable to clinical rehabilitation of pDOC patients. As an objective and reliable evaluation tool, fNIRS possesses important clinical application value in the diagnosis, rehabilitation assessment, and curative effect evaluation of multisensory intervention for pDOC.

## Data Availability

The original contributions presented in the study are included in the article/supplementary material, further inquiries can be directed to the corresponding author.
